# An increase in mean platelet volume during admission can predict the prognoses of patients with pneumonia in the intensive care unit: A retrospective study

**DOI:** 10.1371/journal.pone.0208715

**Published:** 2018-12-11

**Authors:** Ji-Hoon Lee, MinA Park, SeoungWoo Han, Jae Joon Hwang, So Hee Park, So Young Park

**Affiliations:** 1 Department of Pulmonary and Critical Care Medicine, Asan Medical Center, University of Ulsan College of Medicine, Seoul, Korea; 2 Department of Pulmonology, Cheonan Chungmu Hospital, Cheonan, Korea; 3 Division of Pulmonary and Critical Care Medicine, Department of Internal Medicine, Kyung Hee University Medical Center, Seoul, Korea; 4 Department of Internal Medicine, College of Medicine, Kyung Hee University, Seoul, Korea; 5 Department of Pulmonary and Critical Care Medicine, Inje University Ilsan Paik Hospital, Ilsan, Korea; 6 Department of Pulmonary and Critical Care Medicine, Chugnam National University Medical College and Hospital, Daejeon, Korea; University of Notre Dame Australia, AUSTRALIA

## Abstract

Platelets play an important role in hemostasis, inflammation, and immunity. Mean platelet volume (MPV), considered a marker of platelet function and activation, is associated with increased morbidity and mortality in sepsis, coronary artery disease, and chronic inflammatory disease. However, the clinical characteristics and prognostic significance of MPV changes for patients with pneumonia in the intensive care unit (ICU) have not been investigated. This retrospective study was conducted using data from an operational database of patients admitted to a medical ICU between October 2010 and October 2017. Of 235 adult patients with pneumonia admitted to the ICU, clinical characteristics and in-hospital mortality values were compared according to MPV, ΔMPV_day1–2_, ΔMPV_day1–3_, ΔMPV_day1–4_, and ΔMPV_day1–Discharge_ between those who survived and those who did not. The MPV increased during the first four days for both non-survivors and survivors (P < 0.001). However, repeated measures analysis of variance revealed a significantly higher MPV rate over the first four days in non-survivors than in survivors. Additionally, the ΔMPV_day1–2_, ΔMPV_day1–3_, ΔMPV_day1–4_, and ΔMPV_day1–Discharge_ values were significantly greater in non-survivors than in survivors. For in-hospital mortality, the optimal ΔMPV values were >0.9 fL, *P* = 0.020; >0.9 fL, *P* < 0.001; >0.8 fL, *P* < 0.001; and >1.3 fL, *P* < 0.001 on day 2, day 3, day 4, and at discharge, respectively. In conclusion, our findings demonstrate that ΔMPV during ICU admission may be used as a prognostic marker of mortality in ICU patients with pneumonia. Repeated MPV measurements throughout hospitalization may improve risk stratification for these patients, which could aid in improving patient outcomes.

## Introduction

Pneumonia remains the leading cause of death in critical care patients, despite the implementation of several strategies over the last few decades that have aimed to optimize outcomes for patients with pneumonia [1–3]. Early recognition and risk stratification are necessary to improve outcomes for pneumonia patients [4]. However, no factors have been identified to date that show sufficient accuracy as prognostic markers in pneumonia [5].

Platelets are conventionally considered to have a role in hemostasis and thrombosis, but have received increasing attention for their role in inflammation and immune responses [6, 7]. Platelet structure and function are dynamic and can change dramatically when platelets transition from quiescent circulating platelets to activated platelets in response to physiological and pathological signals [8].

Mean platelet volume (MPV) is an indicator of platelet size and activity, and is easily and cost-effectively measured with the use of automated hematology analyzers. An increased platelet volume and size reflect a thrombotic and inflammatory milieu; thus, MPV has been proposed as a possible marker of platelet function and activation [9, 10]. Several studies have reported that MPV changes are associated with morbidity and mortality in patients with diseases such as sepsis, diabetes mellitus, myocardial infarction, and chronic inflammatory disorders [11–14]. One study that had been conducted in intensive care units (ICUs) reported that an elevated MPV was related to a poor prognosis [15].

We hypothesized that the MPV may reflect platelet activity and may be associated with an impaired host response. According to our hypothesis, an increasing MPV may be associated with poor outcomes, and may predict in-hospital mortality in patients in the ICU with severe pneumonia. To test our hypothesis, we examined MPV alterations in patients with severe pneumonia who had been admitted or transferred to the ICU.

## Materials and methods

### Study population and design

This retrospective study was conducted using data from an operational database of patients admitted to the medical ICU of the Kyung Hee University Hospital (a university-affiliated, tertiary referral hospital in Seoul, Korea) between October 2010 and October 2017.

Patients were included if they had been admitted or transferred to the ICU with pneumonia. Patients were excluded from this study if they were <19 years old, or had been admitted to the ICU for <72 hours. In terms of primary hematological disorders, those involving advanced malignant disease, severe thrombocytopenia on admission (platelet count ≤20 × 10^9^/L), and receipt of platelet transfusion during admission were also excluded. This study was reviewed and approved by the institutional review board of Kyung Hee University (IRB No. KHUH 2017-12-011-002). The need for informed consent was waived due to the retrospective nature of the study.

### Data collection

The study participants’ epidemiologic and clinical data were obtained from their medical records. Data included age, sex, comorbid conditions, illness severity scores, laboratory values, and therapeutic interventions such as vasopressor use, renal replacement therapy, or intubation. Samples of peripheral blood were collected using vacutainer blood collection tubes coated with ethylene diamine tetraacetic acid. A complete blood cell count and MPV values were determined using an automated hematology analyzer (ADVIA 2120 and ADVIA 2120i, Siemens, Munich, Germany).

### Definitions

Pneumonia was defined as the presence of at least one of the following: fever (body temperature >38.2°C) or hypothermia (temperature <35.0°C), new cough with or without sputum production, dyspnea or altered breathing sounds on auscultation, presence of a new chest radiographic infiltrate, and no clear evidence of alternative diagnoses, as previously described [16, 17]. Illness severity was determined using acute physiology and chronic health evaluation II (APACHE II) and sequential organ failure assessment (SOFA) scores. Day 1 was defined as the interval from ICU admission to 5:00 am on the next day; all other days were calendar days from 5:00 am to 4:59 am. Sepsis and septic shock were defined as described in the 1991 American College of Chest Physicians/Society of Critical Care Medicine Consensus Conference guidelines [18]. Although these criteria are no longer used, lactic acid levels could not be determined for many of the enrolled patients in this study; hence, the latest definition of sepsis could not be applied.

### Statistical analysis

Mean values and standard deviations were used to summarize the results of the continuous variables, and numbers and percentages were used to summarize the results of the categorical data. Independent two-group comparisons were performed based on the Fisher’s exact test or the χ^2^ test for categorical data, and the Student’s *t*-test for continuous data. Changes in the platelet count and MPV during the first four days after ICU admission and during hospitalization were compared between patients who survived and those who died using repeated measures analysis of variance (rANOVA). The factors associated with survival were determined using Cox regression analysis. Covariate selection for the multivariate Cox model was based on a *P*-value <0.2 in the univariate analysis. Patients were censored if they were discharged or transferred, and the date of discharge or transfer was used as the censoring time. Kaplan-Meier survival curves were produced using in-hospital mortality data, based on the ΔMPV_day1–2_, ΔMPV_day1–3,_ ΔMPV_day1–4_, ΔMPV_day1–Discharge_ and ΔPlatelet_day1–Discharge_ values. Most of the statistical results were obtained using SPSS for Windows (version 20.0, IBM Corporation, Chicago, IL, USA). Kaplan-Meier survival curves were drawn using cut-off points with the MaxStat package of R software.

## Results

### Baseline clinical characteristics

**Fig 1:**
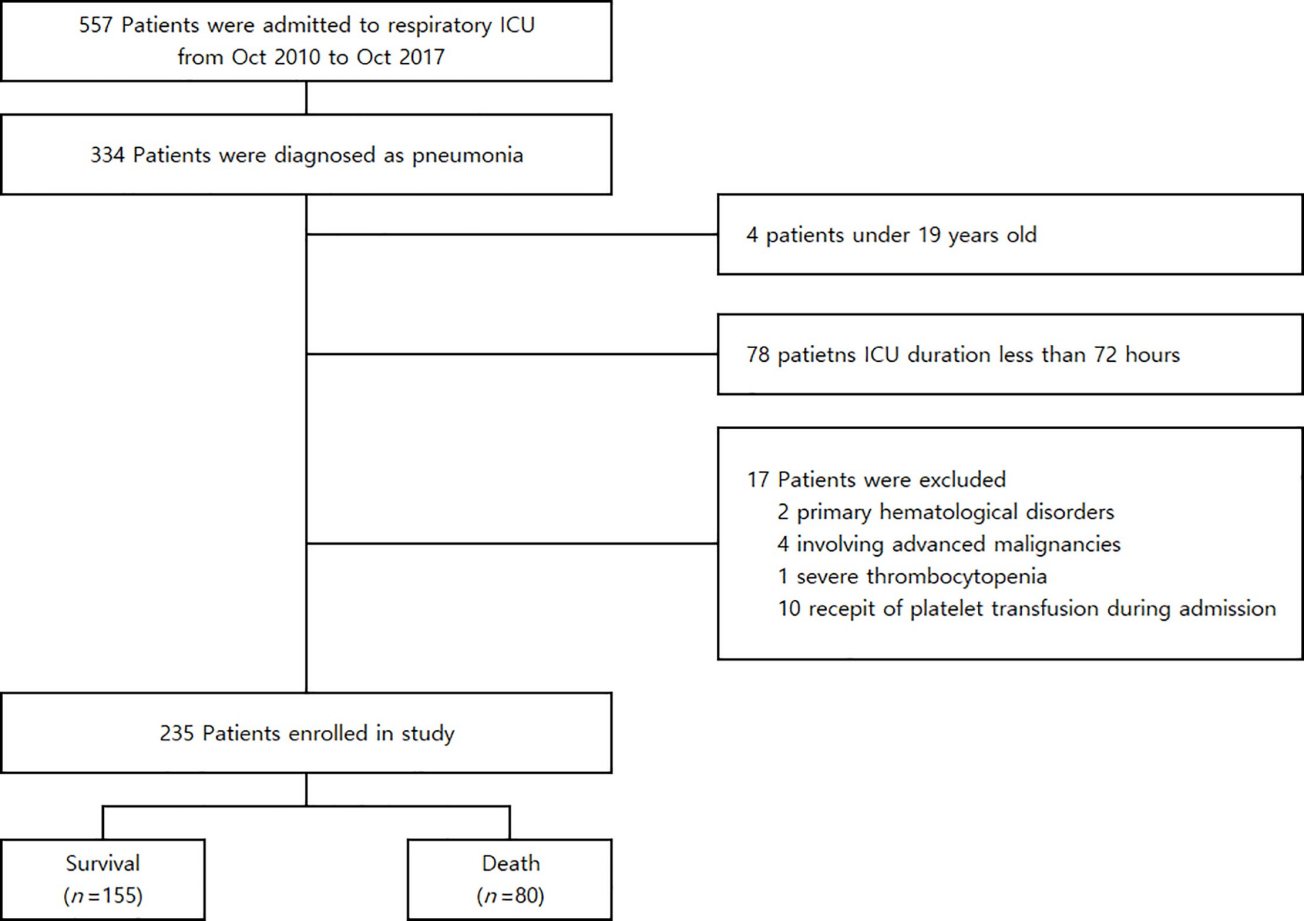
Study design and outcomes.

Of 557 patients admitted to the ICU from October 2010 to October 2017, 235 were enrolled in the study (Fig 1). The mean patient age was 70.5 ± 12.8 years, and 109 (70.3%) were male. The mean APACHE II score was 19.6 ± 6.5, and the mean SOFA score was 7.9 ± 2.9. The baseline demographic, clinical, and biochemical data of each group, stratified according to in-hospital mortality, are presented in Table 1. There were no significant differences in the isolated MPV and platelet count values between the survival and non-survival groups at admission.

**Table 1:** Baseline characteristics of the patients according to in-hospital mortality.

	In-hospital mortality			
	Total	Survival	Death	*P-*value
Variables	(n = 235)	(n = 155)	(n = 80)	
**Demographic data**				
**Sex (male)**	167 (71.1%)	109 (70.3%)	58 (72.5%)	0.844
**Age (years)**	72.3 ± 11.9	70.5 ± 12.8	75.8 ± 9.0	<0.001
**Hospital-acquired pneumonia**	46 (19.6%)	19 (12.3%)	27 (33.8%)	<0.001
**Underlying diseases**				
**Diabetes mellitus**	65 (27.7%)	47 (30.3%)	18 (22.5%)	0.264
**Hypertension**	137 (58.3%)	91 (58.7%)	46 (57.5%)	0.969
**History of malignancy**	34 (14.5%)	21 (13.5%)	13 (16.2%)	0.717
**Congestive heart failure**	17 (7.2%)	7 (4.5%)	10 (12.5%)	0.048
**COPD**	20 (8.5%)	14 (9.0%)	6 (7.5%)	0.879
**Liver cirrhosis**	2 (0.9%)	2 (1.3%)	0 (0.0%)	0.786
**Clinical outcomes**				
**Intubation**	165 (70.2%)	98 (63.2%)	67 (83.8%)	0.002
**CRRT**	4 (1.7%)	2 (1.3%)	2 (2.5%)	0.883
**Vasopressor/inotrope use**	116 (49.4%)	68 (43.9%)	48 (60.0%)	0.027
**Sepsis**	205 (87.2%)	131 (84.5%)	74 (92.5%)	0.126
**Septic shock**	83 (35.3%)	50 (32.3%)	33 (41.2%)	0.221
**ICU length of stay (days)**	18.0 ± 21.2	15.6 ± 21.1	22.6 ± 20.6	0.015
**Clinical data**				
**Mean blood pressure (mmHg)**	88.7 ± 19.7	88.0 ± 20.1	90.0 ± 19.0	0.456
**Heart rate (rate/min)**	105.9 ± 23.3	103.6 ± 21.6	110.5 ± 25.7	0.032
**Respiratory rate (rate/min)**	26.8 ± 7.5	26.0 ± 7.5	28.3 ± 7.3	0.028
**Body temperature (°C)**	36.5 ± 0.8	36.5 ± 0.8	36.5 ± 0.8	0.629
**APACHE II score**	20.6 ± 6.8	19.6 ± 6.5	22.7 ± 6.9	0.001
**SOFA score**	8.3 ± 2.9	7.9 ± 2.9	9.1 ± 2.7	0.002
**Laboratory results**				
**pH**	7.4 ± 0.1	7.4 ± 0.1	7.4 ± 0.1	0.185
**PCO2 (mmHg)**	41.5 ± 16.1	41.5 ± 16.1	41.3 ± 16.3	0.919
**PaO2 (mmHg)**	78.7 ± 23.7	77.2 ± 21.1	81.5 ± 28.0	0.232
**HCO3- (mmol/L)**	22.7 ± 6.0	23.0 ± 6.1	22.0 ± 5.7	0.217
**sO2 (%)**	93.5 ± 4.8	93.6 ± 3.6	93.1 ± 6.5	0.498
**Total bilirubin (mg/dL)**	0.8 ± 1.0	0.8 ± 1.1	0.8 ± 0.6	0.964
**Albumin (g/dL)**	3.3 ± 0.6	3.3 ± 0.6	3.1 ± 0.5	0.036
**C-reactive protein (mg/dL)**	16.7 ± 10.3	16.0 ± 10.1	17.9 ± 10.6	0.183
**Blood urea nitrogen (mg/dL)**	32.5 ± 25.9	30.0 ± 24.3	37.4 ± 28.1	0.038
**Creatinine (mg/dL)**	1.4 ± 1.3	1.4 ± 1.4	1.4 ± 1.2	0.835
**Sodium (mmol/L)**	136.9 ± 6.6	137.0 ± 6.4	136.7 ± 7.2	0.744
**Potassium (mmol/L)**	4.0 ± 0.8	4.0 ± 0.8	4.0 ± 0.8	0.759
**White blood cell (/㎕)**	13.3 ± 7.7	12.9 ± 7.6	14.0 ± 8.0	0.322
**Hemoglobin (g/dL)**	11.6 ± 2.2	11.7 ± 2.0	11.6 ± 2.5	0.783
**Hematocrit (%)**	35.3 ± 6.4	35.4 ± 5.8	35.2 ± 7.3	0.851
**Platelet (10**^**3**^**/㎕)**	250.2±135.3	260.5±146.8	230.1±107.7	0.072
**MPV (fL)**	8.2 ± 1.2	8.2 ± 1.1	8.3 ± 1.5	0.452
**ΔMPV**_**day1-2**_ **(fL)**	0.2 ± 0.7	0.1 ± 0.7	0.3 ± 0.7	0.010
**ΔMPV**_**day1-3**_ **(fL)**	0.3 ± 0.7	0.2 ± 0.7	0.6 ± 0.8	<0.001
**ΔMPV**_**day1-4**_ **(fL)**	0.4 ± 0.9	0.3 ± 0.8	0.7 ± 1.0	0.001
**ΔMPV**_**day1-discharge**_ **(fL)**	0.6 ± 1.8	-0.1 ± 1.1	1.8 ± 2.3	<0.001
**ΔPlatelet**_**day1-2**_ **(10**^**3**^**/㎕)**	-28.1 ± 52.3	-29.8 ± 51.4	-24.8 ± 54.4	0.491
**ΔPlatelet**_**day1-3**_ **(10**^**3**^**/㎕)**	-45.0 ± 68.1	-42.7 ± 71.5	-49.4 ± 61.1	0.480
**ΔPlatelet**_**day1-4**_ **(10**^**3**^**/㎕)**	-48.1 ± 84.7	-42.1 ± 88.5	-59.8 ± 76.0	0.130
**ΔPlatelet**_**day1-discharge**_ **(10**^**3**^**/㎕)**	-9.1 ± 159.9	32.7 ± 154.1	-90.0± 139.2	<0.001

APACHE, acute physiology and chronic health evaluation; COPD, chronic obstructive pulmonary disease; CRRT, continuous renal replacement therapy; ICU, intensive care unit; MPV, mean platelet volume; PaO2, partial pressure of oxygen in arterial blood; PCO2, carbon dioxide partial pressure; SOFA, sequential organ failure assessment

ΔMPV_day1–2,_ the difference between day 1 and day 2 MPV; ΔMPV_day1–3_, the difference between day 1 and day 2 MPV; ΔMPV_day1–4_, the difference between day 1 and day 4 MPV; ΔMPV_day1–Discharge,_ the difference between day 1 and discharge day MPV; ΔPlatelet_day1-2,_ the difference between day 1 and day 2 platelet count; ΔPlatelet_day1-3,_ the difference between day 1 and day 3 platelet count; ΔPlatelet_day1-4,_ the difference between day 1 and day 4 platelet count and; ΔPlatelet_day1-discharge,_ the difference between day 1 platelet count and discharge platelet count

### Trends in the MPV indices during admission

Of 235 pneumonia patients, 58 patients died (mortality rate, 34.0%). The age, heart rate, respiratory rate, albumin, blood urea nitrogen levels, and the APACHE II and SOFA scores at ICU admission were significantly different between the survival and non-survival groups (Table 1).

**Fig 2:**
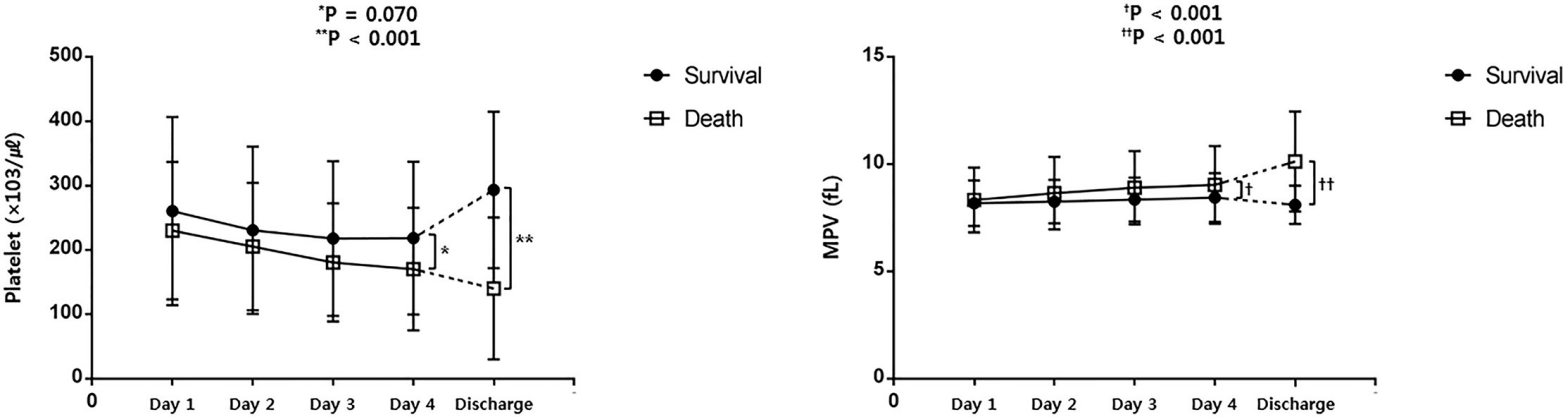
Comparison of the trends in platelet indices between survivors and non-survivors. *Comparison of the day 4 platelet counts between survivors and non-survivors. **Comparison of the discharge day platelet counts between survivors and non-survivors. ^†^Comparison of the day 4 MPV between survivors and non-survivors. ^††^Comparison of the discharge day MPV between survivors and non-survivors.

Trends in platelet indices during the first four days after ICU admission and at discharge are shown in Fig 2. The MPV increased during the first four days in both non-survivors and survivors (*P* < 0.001). However, rANOVA revealed a significantly higher MPV rate over the first four days in non-survivors than in survivors (Fig 2). In addition, the ΔMPV_day1–2_, ΔMPV_day1–3,_ ΔMPV_day1–4_, and ΔMPV_day1–Discharge_ values were significantly greater in non-survivors than in survivors (Table 1). In contrast, although the platelet count decreased significantly during the first four days in both groups (*P* < 0.001), rANOVA showed no significant difference in the platelet count decline rate over the first four days between the groups (Fig 2). Therefore, the ΔPlatelet_day1–2_, ΔPlatelet_day1–3,_ and ΔPlatelet_day1–4_ values showed no statistical significance.

### MPV indices and in-hospital mortality

Univariate Cox regression analysis revealed that age, APACHE II score, HCO_3_^-^, blood urea nitrogen, ΔMPV_day1–2_, ΔMPV_day1–3_, ΔMPV_day1–4_, and ΔMPV_day1–Discharge_ were significantly associated with an increased risk of in-hospital mortality. In the multivariate analysis, ΔMPV_day1–2_, ΔMPV_day1–3_, ΔMPV_day1–4_ and ΔMPV_day1–Discharge_ remained strong predictors of in-hospital mortality, even after adjusting for age, the APACHE II score, HCO_3_^-^, and blood urea nitrogen levels (Table 2).

**Table 2:** Multivariate Cox regression for in-hospital mortality.

Variables	Odds ratio	95% CI	*P*-value
**ΔMPV**_**day1-2**_	1.68	1.06–2.65	0.026
**ΔMPV**_**day1-3**_	2.12	1.37–3.28	<0.001
**ΔMPV**_**day1-4**_	1.72	1.21–2.46	0.003
**ΔMPV**_**day1-discharge**_	2.69	1.95–3.71	<0.001

CI, confidence interval; MPV, mean platelet volume

Adjusted for age, APACHE II score, HCO_3_^-^_,_ and blood urea nitrogen. ΔMPV_day1–2,_ the difference between day 1 and day 2 MPV; ΔMPV_day1–3,_ the difference between day 1 and day 2 MPV; ΔMPV_day1–4_, the difference between day 1 and day 4 MPV and; ΔMPV_day1–Discharge_, the difference between day 1 and discharge day MPV.

This study showed that the prognostic potential of the MPV trend to be better in less severe groups than in more severe groups. Multivariate Cox regression analysis revealed that ΔMPV_day1–2_, ΔMPV_day1–3_, ΔMPV_day1–4_, and ΔMPV_day1–Discharge_ were independent risk factors for in-hospital mortality in patients with septic shock. In patients without septic shock, only ΔMPV_day1–3_ and ΔMPV_day1–Discharge_ showed statistically significant difference (Table 3).

**Table 3:** Multivariate Cox regression for in-hospital mortality in non-septic/septic shock patients.

**With Septic Shock (*n* = 83)**			
**Variables**	**Odds ratio**	**95% CI**	***P*-value**
**ΔMPV**_**day1-2**_	3.17	1.42–7.07	0.005
**ΔMPV**_**day1-3**_	3.02	1.41–6.48	0.005
**ΔMPV**_**day1-4**_	2.27	1.27–4.06	0.006
**ΔMPV**_**day1-discharge**_	2.25	1.45–3.50	<0.001
**Without Septic Shock (*n* = 152)**			
**Variables**	**Odds ratio**	**95% CI**	***P*-value**
**ΔMPV**_**day1-2**_	1.07	0.60–1.88	0.824
**ΔMPV**_**day1-3**_	1.98	1.10–3.60	0.024
**ΔMPV**_**day1-4**_	1.45	0.91–2.31	0.113
**ΔMPV**_**day1-discharge**_	3.17	2.00–5.02	<0.001

CI, confidence interval; MPV, mean platelet volume

Adjusted for age, Acute Physiology and Chronic Health Evaluation II score, HCO_3_^-^ and blood urea nitrogen. ΔMPV_day1–2_: difference between day 1 and day 2 MPV, ΔMPV_day1–3_: difference between day 1 and day 2 MPV, ΔMPV_day1–4_: difference between day 1 and day 4 MPV, ΔMPV_day1–Discharge_: difference between day 1 and discharge day MPV

**Fig 3:**
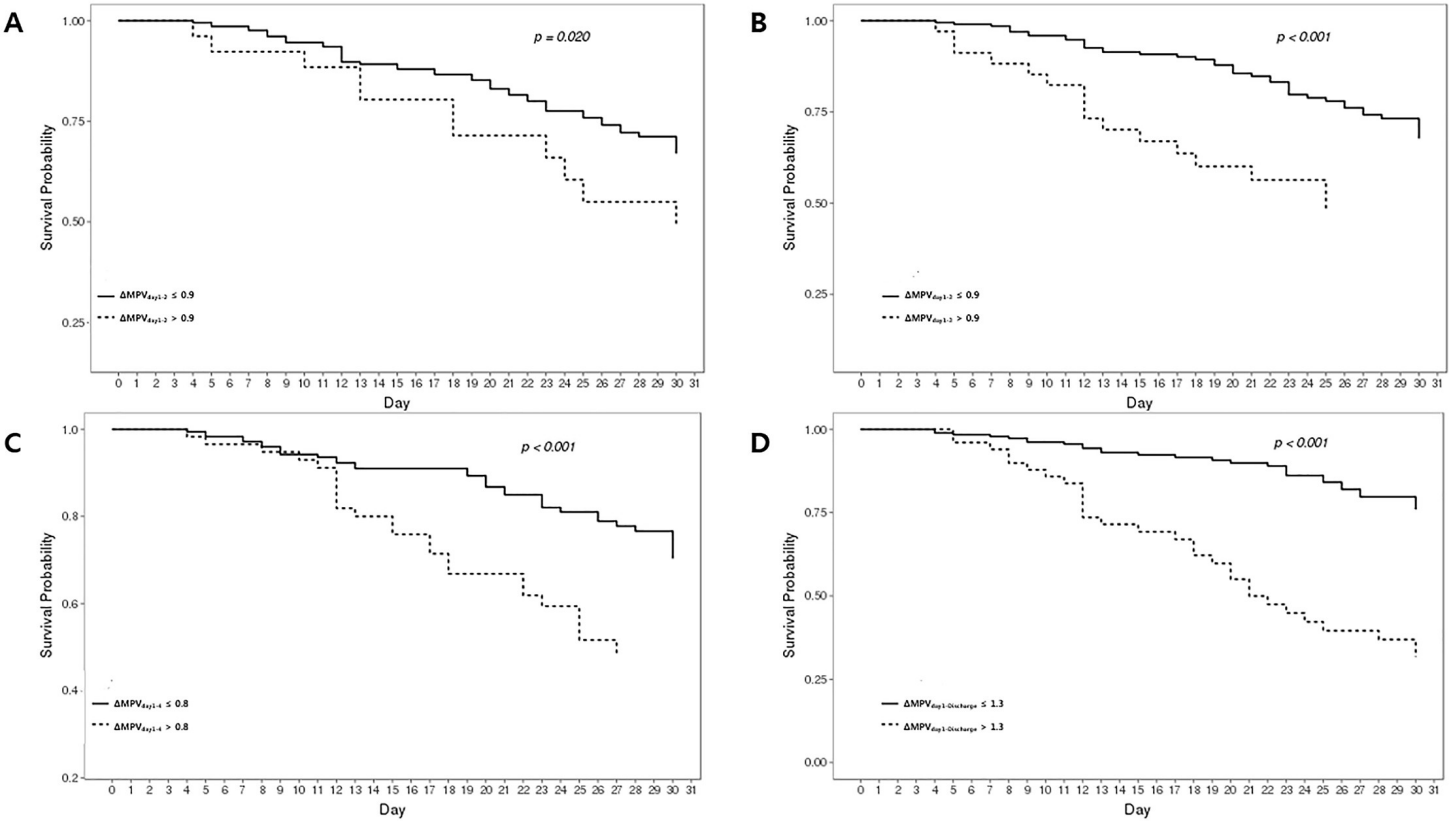
Kaplan-Meier curves for in-hospital mortality. (A) ΔMPV_day1–2_, (B) ΔMPV_day1–3_, (C) ΔMPV_day1–4_ and, (D) ΔMPV_day1–Discharge_.

Based on Kaplan-Meier curves for in-hospital mortality, log-rank tests demonstrated that ΔMPV values were strong predictors (Fig 3). For in-hospital mortality, the optimal ΔMPV values were >0.9 fL, *P* = 0.020; >0.9 fL, *P* < 0.001; >0.8 fL, *P* < 0.001; and >1.3 fL, *P* < 0.001 at day 2, day 3, day 4, and at discharge, respectively.

## Discussion

This study is the first to investigate the association between prognoses and MPV changes in patients with pneumonia in the ICU. Our results indicate that an increasing MPV after ICU admission was significantly associated with poor consequences in our patient population. In addition, non-survivors showed a greater tendency to have a declining platelet count during hospitalization than survivors. Interestingly, we found the prognostic potential of the MPV trend to be better in the sepsis groups than in the septic shock groups.

A recent study showed that the initial MPV, as a predictor of mortality in critically ill patients, was not significantly associated with in-hospital mortality [19]. Another study showed that rising MPV values during hospitalization (MPV on discharge minus MPV on admission) were predictive of in-hospital mortality [20]. However, in that study, the patients had medical conditions of lessor severity than those in our study, and only the differences in the MPV values between admission and discharge were described. We demonstrated the prognostic significance of time-dependent MPV changes. In this study, the MPV at admission was not significantly different between the survivors and non-survivors. However, from day 2 onward, the MPV significantly increased in the non-survivor group, and continued increasing until death. In contrast, in the survivor group, the MPV did not significantly increase until day 4 of admission, and there was no difference or even a slight decline in the value at discharge compared to the MPV at admission. Based on the Kaplan-Meier curves for in-hospital mortality, the log-rank tests demonstrated that ΔMPV values were strong predictors (Fig 3). These data indicate that changes in the MPV rather than the MPV values at admission can provide important information on the disease course.

The mechanism of poor outcomes in pneumonia patients with elevated MPV values in the ICU is unclear. However, we assumed that MPV elevations may reflect hypercoagulation and an increased inflammatory response in pneumonia patients. Since an increased MPV is known to reflect reduced platelet counts and platelet activity [21, 22], the association between increased MPV and poor outcomes in severe pneumonia may suggest that decreasing platelet counts and increasing platelet activity correlate with a distorted host response. Shteinshnaider et al. reported that increases in the platelet counts identified in patients in an inpatient medical ward were associated with better prognoses, and this was more evident in the infectious/inflammatory disease subgroups [23]. These findings suggest that platelet count and MPV changes are related to the inflammatory process as mechanisms associated with disease prognoses. In severe infection, the increased release of thrombopoietin and numerous inflammatory cytokines (e.g., interleukin-1, -3 and -6 and tumor necrosis factor-α) result in increased thrombopoiesis and an enhanced expression of younger large platelets into the bloodstream [10, 24, 25]. Larger platelets contain higher levels of intracellular thromboxane A2 and have an increased expression of procoagulant surface proteins such as p-selectin and glycoprotein IIIa, leading to greater prothrombotic potential. Moreover, platelet neutrophil interaction and platelet endothelial interaction facilitate a variety of immune activation responses [26]. The presence of an increased number of young platelets indicates increased platelet production due to overconsumption induced through inflammation. It may be plausible to assume that the platelets are consumed and even depleted in the immune processes, leading to platelet count declines that are reflected in the MPV as increased platelet activity; however, further investigation is needed to support this speculation.

Previous studies have reported the relationship between elevated MPV and poor prognoses in septic shock [27]. In our participants, elevated MPV values were significantly associated with in-hospital mortality in the presence of septic shock (Table 3). In the absence of septic shock, the in-hospital mortality values increased with an increasing MPV, but the odds ratios were relatively low and there was no statistically significant difference on day 2 and day 4. The odds ratio of MPV changes at discharge was higher than that of septic shock, and statistically significant differences were observed when compared to those in patients without septic shock. This may be attributed to the higher mortality in patients with septic shock (39.8% vs. 30.9%) and the decrease in the elevated MPV in the survivors, thus amplifying the difference. These findings suggest that the mechanism of MPV elevation is related to the infectious/inflammatory process and that the greater the inflammation, the more pronounced the MPV rise.

This study has some limitations. First, all the patients included in this study were enrolled in a single medical center, leading to limitations in the generalizability of the results. One advantage, however, is that all the patients underwent similar procedures for critical care, and were tested using the same equipment. Second, due to the retrospective nature of the study, selection bias or missing data may have distorted the results. Third, a rise in MPV could be related to renal dysfunction and hypoxemia [28, 29]. In this study, we could not evaluate the effect of renal dysfunction and hypoxemia on MPV elevations. Finally, the relatively small sample size we included is a major limitation of this study. A further well-controlled, large-scale prospective study is needed to clarify the relationship between MPV and disease course and the underlying mechanisms.

## Conclusions

In conclusion, the ΔMPV during admission may be used as a powerful predictor of mortality in patients with pneumonia in the ICU. We recommend performing repeated MPV measurements and tracking throughout hospitalization to improve the risk stratification for patients with pneumonia in the ICU.

## Supporting information

S1 File Raw clinical data of all subjects. (ZIP)
